# AHCSIS: adaptive evaluation model for stereoscopic visual comfort based on HHT-CSP-SSA and improved SNN

**DOI:** 10.3389/fnins.2026.1839200

**Published:** 2026-08-11

**Authors:** Yuguo Wang, Jialing Bai, Hui Zhang, Yan Wu

**Affiliations:** 1 Department of Network Construction and Information Management Center, Jilin Business and Technology College, Changchun, China; 2 School of Computer Science and Technology, Changchun University of Science and Technology, Changchun, China; 3 Department of Computer Science and Technology, Zhongshan Institute of Changchun University of Science and Technology, Zhongshan, China

**Keywords:** common spatial pattern, HHT, SNN, sparrow search algorithm, visual comfort

## Abstract

**Introduction:**

To address the issues of feature redundancy in the feature extraction process, as well as the fact that the simulation of biological neural activity remains limited in the SNN encoding layer and the lack of temporal information in the SNN encoding layer, the study proposes an adaptive evaluation model for stereoscopic visual comfort based on HHT-CSP-SSA and improved SNN (AHCSIS).

**Methods:**

The model includes two innovative core modules. The feature extraction module of AHCSIS utilizes the Hilbert-Huang Transform (HHT) combined with Common Spatial Pattern (CSP) to adaptively extract time-frequency features from multiple brain regions, with optimal feature selection achieved using the Sparrow Search Algorithm (SSA). The classification module employs the spike encoding method based on the self-attention mechanism combined with a single neuron to generate temporal spike sequences that represent biological neural activity.

**Results:**

The results demonstrate that the performance of the AHCSIS classification model (accuracy: 93.86%, precision: 93.12%) surpasses that of baseline and state-of-the-art models.

**Discussion:**

The model adaptively extracts optimal features and encodes temporal spikes, which better reflect biological neural activity, providing a novel solution for evaluating stereoscopic visual comfort and discomfort.

## 1 Introduction

With the rapid advancement of 3D technology, this technology has been widely applied in fields including aerospace, education, and healthcare (Battulga et al., 2012). However, long-term viewing of stereoscopic images can cause visual discomfort, such as eye fatigue and dizziness (Karatas, 2008). Therefore, it is necessary to evaluate visual discomfort to facilitate the advancement of 3D technology. Electroencephalogram (EEG) has high temporal resolution and can record millisecond-level brain activities (Ford et al., 2021), making it an effective method for objectively evaluating stereoscopic visual discomfort (Zheng et al., 2022).

Feature extraction is crucial in the evaluation process as it affects the accuracy of the results. The common spatial pattern (CSP) method is widely applied in feature extraction for stereoscopic visual comfort (Kang et al., 2017). It utilizes matrix diagonalization to identify optimal spatial filters for projection during feature extraction, maximizing the variance difference between two signal types and producing highly differentiated feature vectors (Alizadeh and Omranpour, 2023; Mishuhina and Jiang, 2021; Liu et al., 2023). Miao et al. (Miao et al., 2021) proposed the Common Time-Frequency Spatial Pattern (CTFSP) method to extract sparse CSP features from multi-band filtered EEG data across multiple time windows. Mishuhina et al. (Mishuhina and Jiang, 2021) proposed a Time-Frequency Common Spatial Pattern (TFCSP) method, which divides the signal of each task into ten frequency groups and then applies complex CSP to extract time-frequency features. Hu et al. (Hu et al., 2022) proposed a novel circulant singular spectrum analysis embedded CSP (CiSSA-CSP) method, in which the original EEG data is divided into multiple time periods, and the CiSSA-CSP method is used to further extract the sub-band information from the non-overlapping frequency bands of each time period. Most CSP-related studies use fixed frequency divisions, but individual differences exist in the frequency bands associated with brain oscillations (Higashi and Tanaka, 2013; Lee et al., 2009; Vossen et al., 2015). Consequently, fixed frequency divisions may fail to capture different participants' variations and transient fluctuations in oscillatory patterns, ultimately reducing classification model accuracy (Shan et al., 2021; Zheng et al., 2022).

Some studies have utilized the adaptive properties of the Hilbert-Huang transform (HHT) to divide the frequency bands of EEG signals, enabling more accurate capture of the hidden oscillatory activities of EEG signals from different participants (Zheng et al., 2022; Obata et al., 2023). Zheng et al. (Zheng et al., 2022) used HHT to calculate specific frequency bands of brain oscillations, lifting the restriction of using fixed frequency bands. (Anuragi et al., 2020) extracted statistical features, such as mean and standard deviation, from each intrinsic modal function (IMF) using the empirical wavelet transform (EWT) based on HHT (Alizadeh and Omranpour, 2023). During Empirical Mode Decomposition (EMD), a key step in HHT, EEG signals often experience significant fluctuations at temporal boundaries (the beginning and end of the signal), which propagate inward and disrupt the overall signal, ultimately leading to undesirable IMFs (Yedukondalu and Sharma, 2023; Kamble and Sengupta, 2022). These undesirable IMFs lead to redundancy in time-frequency features (Xu et al., 2023), impacting classification performance. Therefore, it is necessary to select optimal time-frequency features to reduce the impact of redundancy on performance. The Sparrow Search Algorithm (SSA) (Gharehchopogh et al., 2023) is an efficient method for exploring and identifying optimal feature combinations within a multidimensional feature space, simulating sparrow behaviors of foraging, anti-predation, and warning to reduce feature redundancy (Gad et al., 2022). Gad et al. (Gad et al., 2022) used Improved Binary SSA (iBSSA) to select optimal or near-optimal features and achieve high classification accuracy. Inspired by the SSA, Chang et al. (Chang et al., 2023) introduced the Swarm Intelligence Optimization Algorithm (SIOA), which enhances search efficiency through a random walk strategy, selecting the best discriminative features and effectively reducing feature redundancy. Therefore, it is speculated that using HHT during the feature extraction phase can adaptively extract time-frequency features, and applying SSA can select the optimal time-frequency feature combinations, with both working together to provide data support for classification models.

In the process of stereoscopic visual comfort classification, Artificial Neural Networks (ANN) have been widely used due to their powerful feature learning and classification capabilities (Wu and Feng, 2018). ANN simulates the basic structure of biological neural networks but still differs from real biological neural activity (Agatonovic-Kustrin and Beresford, 2000). Spiking Neural Networks (SNN), a type of third-generation neural network, exhibit rich spatiotemporal neurodynamics and diverse coding mechanisms (Ghosh-Dastidar and Adeli, 2009). These characteristics enable SNN to closely resemble biological neuron models and bridge the gap between neuroscience and learning algorithms (Virgilio et al., 2020). As a result, some studies have used SNN to classify EEG-based tasks, taking advantage of its ability to mimic real biological neural activity. Liu et al. (Liu et al., 2023) used spike attention coding (SAC) to learn optimal coefficients, solving the coding rate delay problem and improving SNN performance. Liao et al. (Liao et al., 2023) used learnable adaptive coding to reduce information loss and addressed SNN training difficulty through surrogate gradient learning, improving accuracy in motor imagery classification tasks. Virgilio et al. (Virgilio et al., 2020) achieved high accuracy in motor imagery classification using a single-layer neuron model and an SNN model with a multilayer perceptron (MLP) structure, demonstrating that better results can be achieved with fewer neurons. Li et al. (Li et al., 2024) proposed a hybrid response model for SNN-based architectures (HR-SNN) that uses parametric gradient descent to optimize spike encoding efficiency, with the model performing well on motor imagery tasks. In SNN models, neural activity is transmitted through spikes, necessitating spike encoding of the raw signal (Xu et al., 2024). Spikes in current SNN models are typically encoded using Poisson encoding (Guo et al., 2019), which has a certain randomness and may lead to loss of original data, preventing SNN from receiving complete neural activity information (Liao et al., 2023). Moreover, these methods typically overlook the critical role of “time” in the encoding process, leading to extended inference times (Rathi and Roy, 2023). Therefore, a method should be proposed that maintains the accuracy of the original data while effectively containing rich temporal information in the spike encoding process. Since the self-attention mechanism captures temporal information in EEG by calculating attention between different time points in the sequence (Tao et al., 2023), and fewer neurons can maintain sufficient data accuracy when processing signals related to biological neural activity (Virgilio et al., 2020; Liao et al., 2023), the spike encoding method in SNN can be improved by combining the self-attention mechanism with a single neuron.

This study proposes an adaptive evaluation model for stereoscopic visual comfort based on HHT-CSP-SSA and improved SNN (AHCSIS), which consists of two innovative modules. The feature extraction module uses HHT and SSA methods to adaptively divide frequency bands and select optimal feature combinations, comprehensively capturing features of stereoscopic visual comfort and discomfort. In the classification module, a spike encoding method based on the self-attention mechanism combined with a single neuron is used to obtain high-precision temporal spike sequences. The main contributions of this paper are as follows:

1. Based on the CSP method, combined with HHT to adaptively extract time-frequency features and solve the problem of fixed frequency band division.
2. Optimal time-frequency feature combinations are selected using the SSA method to reduce redundant features.
3. A spike encoding method based on the self-attention mechanism combined with a single neuron is used to obtain temporal spike sequences with biological neural activity in SNN.

## 2 Materials and methods

**Figure 1:**
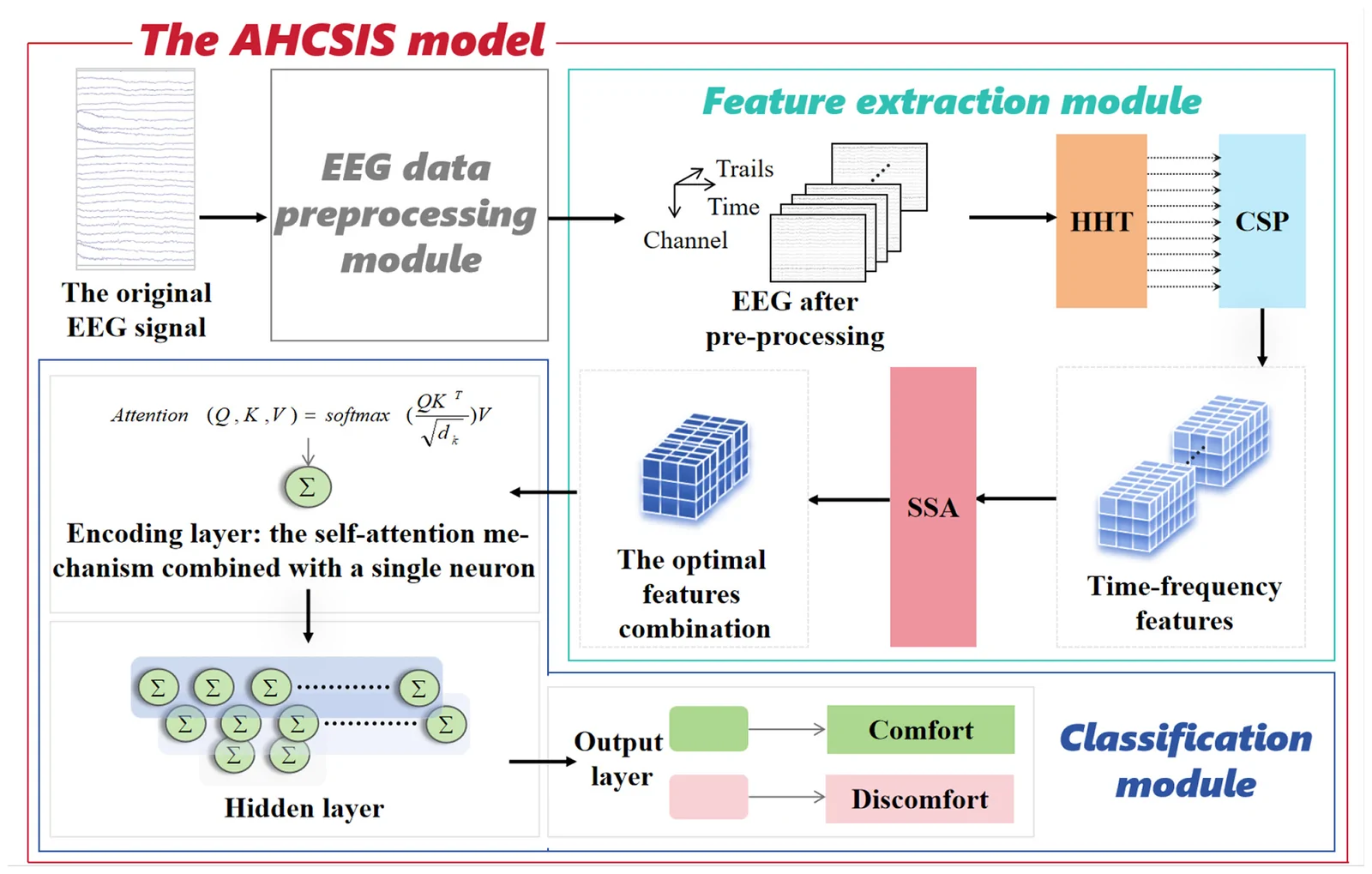
Structure of the AHCSIS model. The AHCSIS model includes EEG data pre-processing module, feature extraction module with HHT, CSP, and SSA, and using the spike encoding method based on the self-attention mechanism combined with a single neuron in the encoding layer of the classification module.

The architecture of the AHCSIS model developed in this study for stereoscopic visual comfort evaluation is shown in Figure 1. The model consists of three main modules: EEG data pre-processing module, feature extraction module, and classification module.

### 2.1 EEG data pre-processing module

In this study, to obtain clean EEG signals, the raw EEG data were processed through filtering, segmentation, baseline correction, and artifact removal. (1) Filtering: a digital filter was applied to eliminate high-frequency noise and low-frequency drift from the EEG signals. The filtering range was set to 0.01–40 Hz, covering the primary frequency band associated with stereoscopic visual comfort-related EEG signals. (2) Segmentation and baseline correction: the –200 to 800 ms time window was segmented, using the –200 to 0 ms period (before the appearance of the stereoscopic image) as the baseline for baseline correction. (3) Artifact removal: EEG data are often contaminated by external factors, including eye and head movements. In this study, Independent Component Analysis (ICA) (Wang et al., 2015), an open-source toolbox available on the Matlab platform (Delorme and Makeig, 2004), was used to remove these irrelevant components. Specifically, independent components displaying localized high-frequency topography and high-variance activation profiles characteristic of electromyogram (EMG) or muscle tension artifacts were visually inspected and discarded during reconstruction to ensure signal purity. Furthermore, the physical mouse click action only occurred chronologically outside the analyzed EEG data epochs (–200 ms to 800 ms), completely preventing active motor execution artifacts from contaminating the stimulation response window.

### 2.2 Feature extraction module

**Figure 2:**
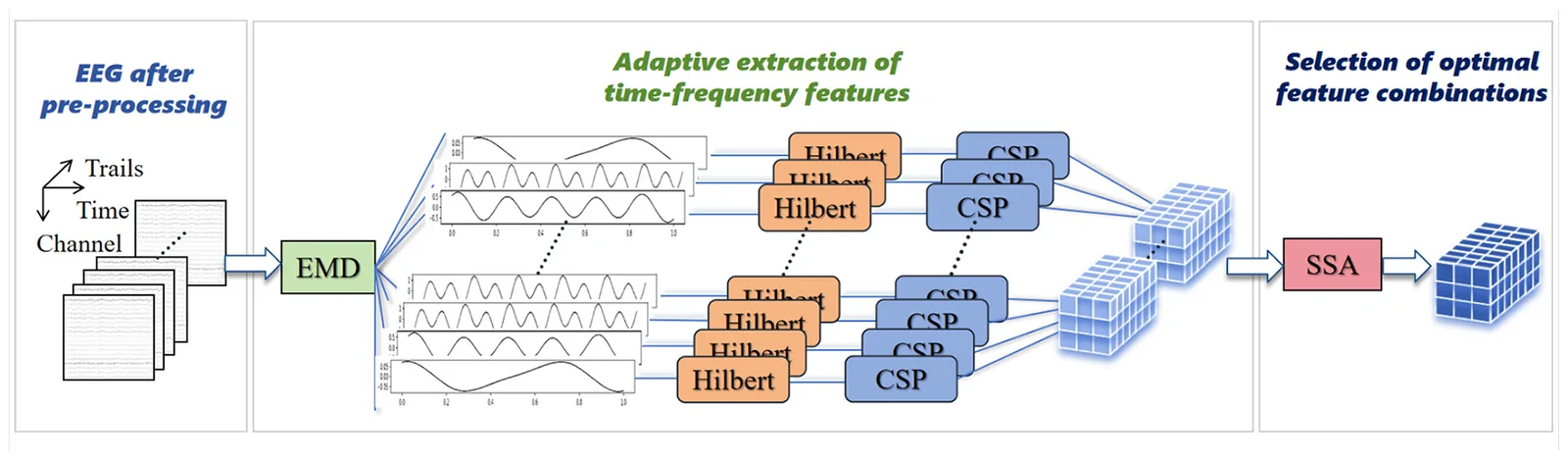
Feature extraction module. Pre-processed EEG signals were decomposed into several IMFs using EMD, with time-frequency features across multiple brain regions adaptively extracted using the Hilbert transform and CSP, and the optimal feature combinations selected using SSA.

As shown in Figure 2, the feature extraction module consists of several key stages. Pre-processed EEG signals are decomposed using EMD to obtain intrinsic mode functions. The Hilbert Transform is applied to adaptively extract time-frequency features, while CSP captures time-frequency features across multiple spatial dimensions. SSA is used to optimize and select the most representative feature combinations for further analysis.

#### 2.2.1 Adaptive extraction of time-frequency features

Time-frequency features across multiple brain regions were adaptively extracted using a combination of the HHT and CSP methods. The HHT consists of EMD and the Hilbert transform. The original EEG signal is decomposed by EMD into a series of IMFs, which represent the oscillatory modes of different frequency components in the signal and offer improved noise resistance (Yang et al., 2020). The Hilbert transform is applied to each IMF to adaptively obtain its instantaneous frequency and amplitude, revealing the dynamic properties of the EEG signal. Moreover, HHT is not constrained by assumptions of linearity or smoothness. This makes it particularly advantageous for processing non-linear and non-smooth signals (Souza et al., 2022).

Decomposition of the EEG signal *X*(*t*) = *C*_*eeg*_ × *T* into a series of IMFs involves deriving the local maxima and minima, using cubic spline interpolation to fit these extreme points. The mean values *X*_0_(*t*) of the upper and lower envelopes of *X*(*t*) are obtained, and the mean value *X*_0_(*t*) is subtracted from the raw EEG signal *X*(*t*) to obtain *h*_1_(*t*) (Equation 1):


$$\begin{array}{l} h_{1}\left(t\right)=X\left(t\right)-X_{0}\left(t\right) \end{array}$$ (1)

For *h*_1_(*t*), the number of extreme points must be equal to the number of zero crossings, or their absolute difference should be one. Additionally, the average of the upper and lower envelopes of *h*_1_(*t*) must be zero. When *h*_1_(*t*) satisfies the above condition for an IMF, the first IMF component is successfully isolated and denoted as *C*_1_(*t*). To iteratively extract subsequent oscillatory modes, the corresponding residual signal *r*_1_(*t*) representing the remaining data sequence is obtained as follows (Equation 2):


$$\begin{array}{l} r_{1}\left(t\right)=X\left(t\right)-C_{1}\left(t\right) \end{array}$$ (2)

The decomposition of the residual signal *r*_1_(*t*) continues until all intrinsic mode functions (IMFs) are successfully extracted. The iterative process stops when *r*_1_(*t*) no longer satisfies the stopping criterion, which is defined by the standard deviation (SD) of the residual signal. The SD is calculated as (Equation 3):


$$\begin{array}{l} SD=\sqrt{\frac{1}{N}\overset{N-1}{\underset{t=0}{\sum }}{\left(r_{1}\left(t\right)-\bar{r_{1}}\right)}^{2}} \end{array}$$ (3)

$\bar{r_{1}}$ is the residual signal mean, and *N* represents the total number of data points in the signal. The decomposition process continues as long as the SD of the residual signal is within the range 0.2 ≤ SD ≤ 0.3. Once this condition is no longer met, the process stops. After extracting all the IMF components, the original EEG signal *X*(*t*) can be fully reconstructed by summing all the derived IMFs and the final residual term *r*_*n*_(*t*), which demonstrates the completeness property of the EMD. The reconstructed mathematical expression is (Equation 4):


$$\begin{array}{l} X\left(t\right)=\overset{n}{\underset{i=1}{\sum }}C_{i}\left(t\right)+r_{n}\left(t\right) \end{array}$$ (4)

Where *C*_*i*_(*t*) denotes the IMF component obtained in the *i*-th iteration, *n* is the total number of iterations, and *r*_*n*_(*t*) is the residual term. After the EMD decomposition, the Hilbert transform is applied to each IMF component to obtain the analytic signal:


$$\begin{array}{l} Y_{i}\left(t\right)=\frac{1}{\pi }\int_{-\infty }^{+\infty }\frac{C_{i}\left(\tau \right)}{t-\tau }d\tau \end{array}$$ (5)

At the same time, the analytical signal is constructed as:


$$\begin{array}{l} Z_{i}\left(t\right)=C_{i}\left(t\right)+jY_{i}\left(t\right)=A_{i}\left(t\right)e^{\theta_{i}\left(t\right)} \end{array}$$ (6)

In this context, is a normalization factor, *C*_*i*_(τ) is the integral variable introduced when performing integration and transformation on the IMF component, 7 is the time variable, and *t* − τ represents the phase shift at different moments. *Z*_*i*_(*t*) is the analytical signal obtained in the i-th iteration, *Y*_*i*_(*t*) is the Hilbert transform of *C*_*i*_(*t*), j is the imaginary unit, *A*_*i*_(*t*) is the instantaneous amplitude obtained in the i-th iteration, and θ_*i*_(*t*) is the instantaneous phase obtained in the i-th iteration.

The instantaneous amplitude and phase are derived from Equations 5, 6 as follows (Equations 7, 8):


$$\begin{array}{l} A_{i}\left(t\right)=\sqrt{{Y_{i}\left(t\right)}^{2}+{C_{i}\left(t\right)}^{2}} \end{array}$$ (7)

$$\begin{array}{l} \theta_{i}\left(t\right)=\arctan\frac{Y_{i}\left(t\right)}{C_{i}\left(t\right)} \end{array}$$ (8)

Then, the instantaneous frequency *W*_*i*_(*t*) is calculated (Equation 9):


$$\begin{array}{l} W_{i}\left(t\right)=\frac{d\theta_{i}\left(t\right)}{dt} \end{array}$$ (9)

By extracting the instantaneous frequency and amplitude of each IMF component, the original EEG signal *X*(*t*) can be reconstructed as (Equation 10):


$$\begin{array}{l} X\left(t\right)=Re\left[\overset{n}{\underset{i=1}{\sum }}A_{i}\left(t\right)e^{j\int W_{i}\left(t\right)dt}\right]+r_{n} \end{array}$$ (10)

Here, Re[] represents the real part of the *X*(*t*) signal, while *r*_*n*_ is the residual, which is non-decomposable by the EMD and can thus be omitted. After applying the HHT, the single-subject time-frequency features *X*(*t*) are categorized into *E*_1_ and *E*_2_, corresponding to stereoscopic visual comfort and discomfort states, respectively.

The basic principle of the CSP method is to find a set of optimal spatial filters through matrix diagonalization to maximize the variance difference between *E*_1_ and *E*_2_ signals, resulting in eigenvectors with a high degree of discrimination. The combination of HHT and CSP enables comprehensive and adaptive extraction of time-frequency features from EEG signals across multiple dimensions. This approach improves the accuracy and reliability of the visual comfort classification model. The covariance matrix for the two feature matrices *E*_1_ and *E*_2_ is computed as follows (Equation 11):


$$\begin{array}{l} C_{i}=\frac{E_{i}\cdot {E_{i}}^{T}}{\mathrm{trace}\left(E_{i}\cdot {E_{i}}^{T}\right)}\;\left(i=1,2\right) \end{array}$$ (11)

Here, ${E_{i}}^{T}$ denotes the transpose of *E*_*i*_ feature matrix, and $trace(E_{i}\cdot E_{i}^{\;T})$ represents the sum of the diagonal elements of the matrix. The mixed spatial covariance matrix is obtained as $C=\bar{C_{1}}+\bar{C_{2}}$. Let $\bar{C_{1}}$ and $\bar{C_{2}}$ represent the expected spatial covariance matrices for the two sample types. Singular value decomposition is used to perform eigenvalue decomposition on the mixed spatial covariance matrix *C*. After whitening transformation, we obtain *C* = *U* Λ *U*^*T*^, where *U*^*T*^ is the transpose of *U*, and *U* is the eigenvector matrix. Apply the whitening eigenvector matrix *P* to *C*_1_ and *C*_2_ (Equations 13, 14):


$$\begin{array}{l} P=\frac{1}{\sqrt{\Lambda }}U^{T} \end{array}$$ (12)

$$\begin{array}{l} S_{1}=PC_{1}P^{T} \end{array}$$ (13)

$$\begin{array}{l} S_{2}=PC_{2}P^{T} \end{array}$$ (14)

S1 and S2 have common eigenvectors, and there exist two diagonal matrices Λ_1_ and Λ_2_ with the same eigenvectors B (Equations 15–17):


$$\begin{array}{l} S_{1}=B\Lambda_{1}B^{T} \end{array}$$ (15)

$$\begin{array}{l} S_{2}=B\Lambda_{2}B^{T} \end{array}$$ (16)

$$\begin{array}{l} \Lambda_{1}+\Lambda_{2}=I \end{array}$$ (17)

The sum of Λ_1_ and Λ_2_ equals the identity matrix. If the eigenvalues of Λ_1_ are ordered in descending order, the eigenvalues of Λ_2_ will be in ascending order. Thus, for eigenvector B, when *S*_1_ has the largest eigenvalue and *S*_2_ the smallest, classification can be achieved using B, resulting in the projection matrix W:


$$\begin{array}{l} W=B^{T}P \end{array}$$ (18)

Here, the projection matrix *W* serves as the corresponding spatial filter. The EEG signals *E*_*i*_(*i* = 1, 2) are first projected through *W* to obtain the feature matrices *Z*_*i*_ = *W* × *E*_*i*_(*i* = 1, 2), which are then normalized to obtain the final features (Equation 19):


$$\begin{array}{l} f_{i}=\frac{Var\left(Z_{i}\right)}{\sum \left(Var\left(Z_{i}\right)\right)} \end{array}$$ (19)

In this case, the variance of the features is calculated using Var, and *f*_*i*_ represents the feature matrix normalized to the i-th sample.

#### 2.2.2 Selection of optimal feature combinations

The SSA has a unique optimization mechanism and strong global search capability (Ma et al., 2021). By combining sparrows' foraging, anti-predation, and early warning behaviors, along with the behavioral patterns of different individuals, SSA can effectively search for and select optimal feature combinations in complex feature spaces. This reduces feature redundancy and enhances the model's classification performance.

Initialization: set the SSA parameters, including population size (number of sparrows), iteration number, and search space range. The sparrow population is randomly initialized, where each sparrow represents a combination of features, and its position is described by a set of eigenvalues. The population consisting of *n* sparrows is represented as (Equation 20):


$$X=\left[\begin{array}{ccc} \begin{array}{cc} X_{1,1} & X_{1,2}\\ X_{2,1} & X_{2,2} \end{array} & \cdots & \begin{array}{c} X_{1,d}\\ X_{2,d} \end{array}\\ \vdots & \cdots & \vdots \\ \begin{array}{cc} X_{n,1} & X_{n,2} \end{array} & \cdots & X_{n,d} \end{array}\right]$$ (20)

*d* denotes the dimension of the variable to be optimized.

Fitness assessment: for each sparrow, the fitness function (classification accuracy or cross-validation score) is used to evaluate the performance of stereoscopic visual comfort classification. The fitness value of each sparrow is then calculated and sorted based on the fitness value. The fitness values for all sparrows are represented as:


$$F_{x}=\left[\begin{array}{ccc} \begin{array}{cc} f([X_{1,1} & X_{1,2}\\ f([X_{2,1} & X_{2,2} \end{array} & \cdots & \begin{array}{c} X_{1,d}])\\ X_{2,d}]) \end{array}\\ \vdots & \cdots & \vdots \\ \begin{array}{cc} f([X_{n,1} & X_{n,2} \end{array} & \cdots & X_{n,d}]) \end{array}\right]$$ (21)

*f* denotes the fitness value.

Role assignment: sparrows are categorized into discoverers, followers, and vigilantes based on their fitness values. Discoverers are sparrows with high fitness values, responsible for leading the search. In contrast, followers have relatively lower fitness values and adjust their positions by following the discoverers. Vigilantes monitor the environment and promptly issue warnings when potential threats are detected to ensure the safety of the group.

Position update: the discoverer focuses on local optimization within the current solution space while also searching a broader area for the global optimal feature combination. The follower dynamically adjusts its position based on the discoverer, allowing it to escape the limitations of the current search area. The vigilante adjusts its search behavior according to environmental safety conditions, adopting strategies to avoid local optima when the environment is unsafe. The position of the discoverer is updated as follows:


$$\begin{array}{l} X_{i,j}^{t+1}=\{\begin{array}{lll} X_{i,j}^{t}\cdot \exp\left(-\frac{i}{a\cdot T}\right) & \text{if} & R_{2}<ST\\ X_{i,j}^{t}+Q\cdot L & \text{if} & R_{2}\geq ST \end{array} \end{array}$$ (22)

Here, *t* represents the current iteration number, *T* is the maximum number of iterations, and *a* ∈ (0, 1] is a random number. The parameters *R*_2_ ∈ [0, 1] and *ST* ∈ [0.5, 1] represent the preset alarm value and safety threshold, respectively; these mathematical bounds are standard behavioral hyper-parameters derived from established benchmarks in the SSA literature (20) to effectively balance the population's safe foraging exploitation and global anti-predation exploration. *Q* is a random number following a normal distribution, and *L* denotes a 1 × *D* matrix.

Follower position updates:


$$\begin{array}{l} X_{i,j}^{t+1}=\{\begin{array}{c} Q\cdot \exp\left(\frac{X_{worst}^{t}-X_{i,j}^{t}}{i^{2}}\right)\;\text{if }i<n/2\\ X_{p}^{t+1}+|X_{i,j}^{t}-X_{p}^{t+1}|\cdot A^{+}\cdot L\;\text{otherwise} \end{array} \end{array}$$ (23)

Here, *X*_*p*_ represents the optimal position currently occupied by the discoverer, while $X_{worst}^{t}$ denotes the current global worst position. *A* is a 1 × *d* matrix where each element is randomly assigned a value of 1 or –1, and *A*^+^ =*A*^*T*^(*AA*^*T*^)^−1^. When *i* > *n*/2, the *i*-th follower with a lower fitness value updates its position.

Anti-predation behavior: an anti-predator mechanism is introduced during the search process. A warning signal is emitted when a potential threat is detected by the vigilante (a sudden drop in fitness value or reaching a preset alarm threshold). Sparrows receiving the warning signal will adjust their search strategy, such as increasing the search step size or changing the search direction, to escape from potential “predators” (local optima).


$$\begin{array}{l} X_{i,j}^{t+1}=\{\begin{array}{ll} X_{best}^{t}+\beta \cdot |X_{i,j}^{t}-X_{best}^{t}| & \text{if }f_{i}>f_{g}\\ X_{i,j}^{t}+K\cdot \left(\frac{X_{i,j}^{t}-X_{worst}^{t}}{\left(f_{i}-f_{w}\right)+\varepsilon }\right) & \text{if }f_{i}=f_{g} \end{array} \end{array}$$ (24)

$X_{best}^{t}$ represents the global optimal position in the current iteration. β is the step control parameter, a random number following a standard normal distribution, referred to as the regulating step factor. *K* ∈ [−1, 1] determines the direction of the sparrow's movement. ε is a small constant to prevent division by zero. *f*_*g*_ and *f*_*w*_ represent the best and worst fitness values in the current population, respectively, and *f*_*i*_ is the fitness value of the i-th sparrow.

**Figure 3:**
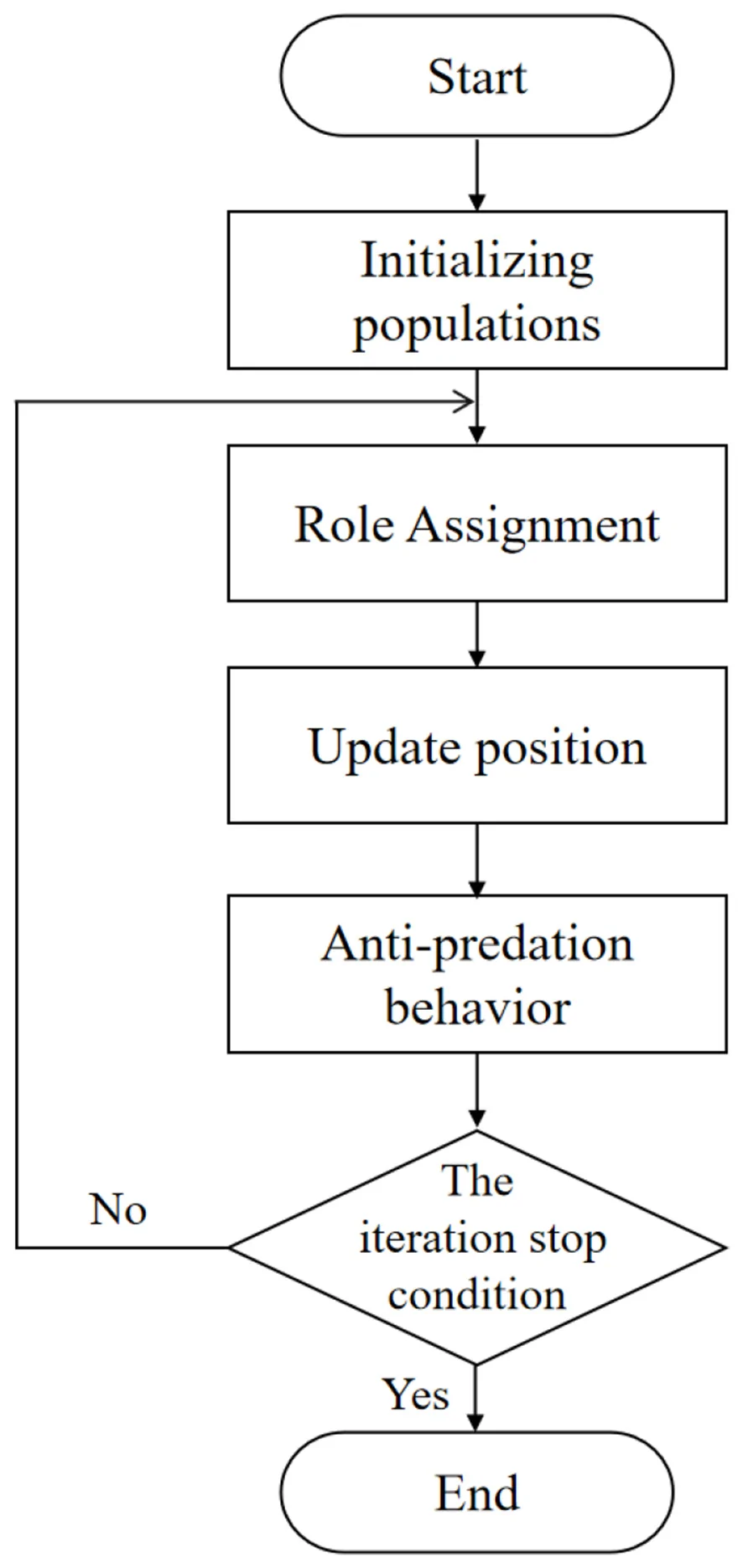
SSA algorithm flow chart. The process begins with population initialization, followed by role assignment and position updates. It incorporates anti-predation behavior and iterates through these steps until the iteration stopping condition is satisfied, marking the end of the algorithm.

Iterative optimization: in each iteration, Equations 21–24 are repeated to optimize feature combinations by continuously updating the positions of the sparrows and their role assignments. The flow chart of the SSA algorithm is shown in Figure 3. As iterations progress, the algorithm gradually converges toward the optimal feature combination. At the end of the iterative process, the optimal sparrow (the optimal feature combination) is selected based on its fitness value, and the performance of this feature combination is validated using an independent validation or test set.

### 2.3 Classification module

**Figure 4:**
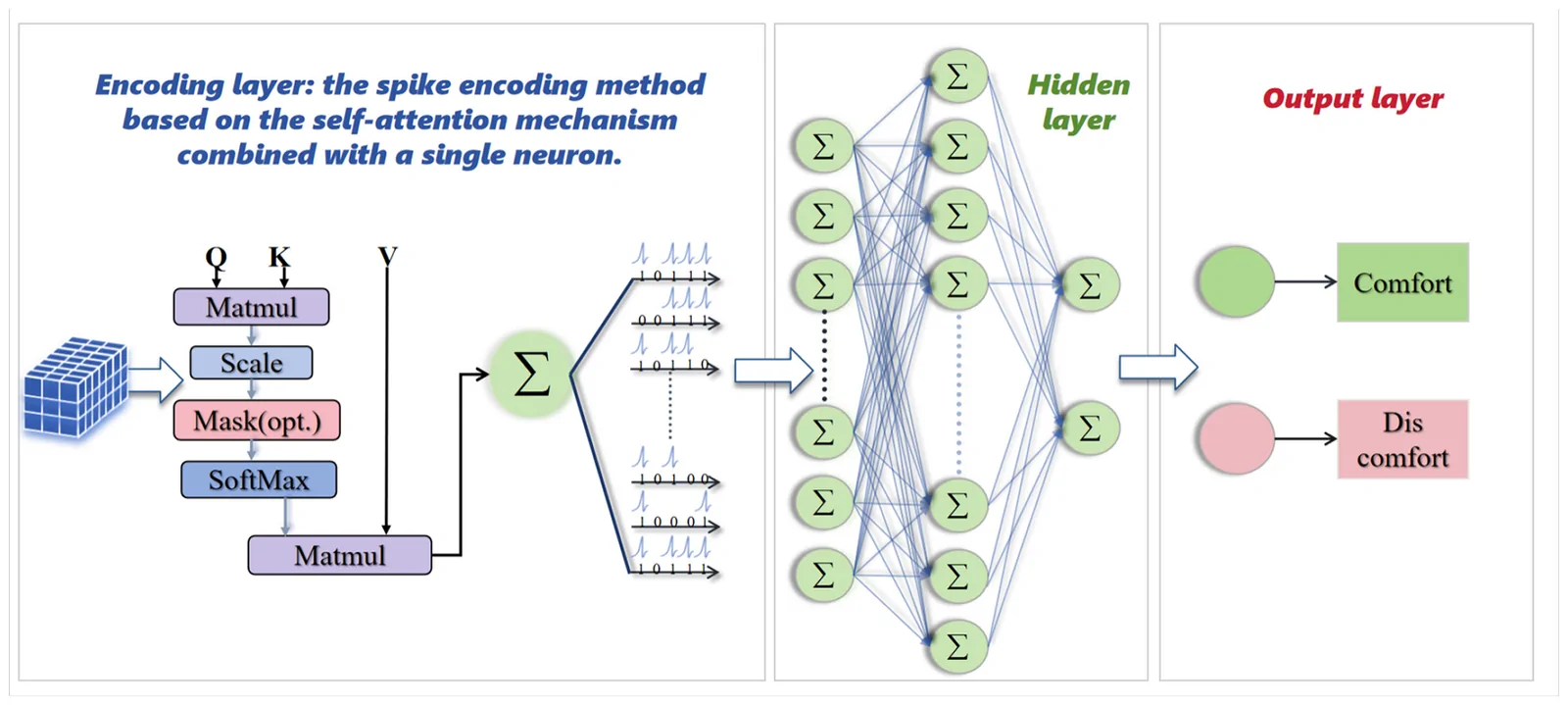
Classification module. The encoding layer is replaced by the self-attention mechanism combined with a single neuron, instead of traditional spike encoding, followed by the hidden layer for feature processing and the output layer that classifies inputs into “comfort” and “discomfort” categories.

The optimal time-frequency features are classified using an SNN, where the encoding layer leverages the spike encoding method based on the self-attention mechanism combined with a single neuron to generate high-precision temporal spike sequences. These sequences undergo advanced processing in the hidden layer and are ultimately decoded into comfort and discomfort categories in the output layer, as illustrated in Figure 4.

#### 2.3.1 Encoding layer: the spike encoding method based on the self-attention mechanism combined with a single neuron

**Figure 5:**
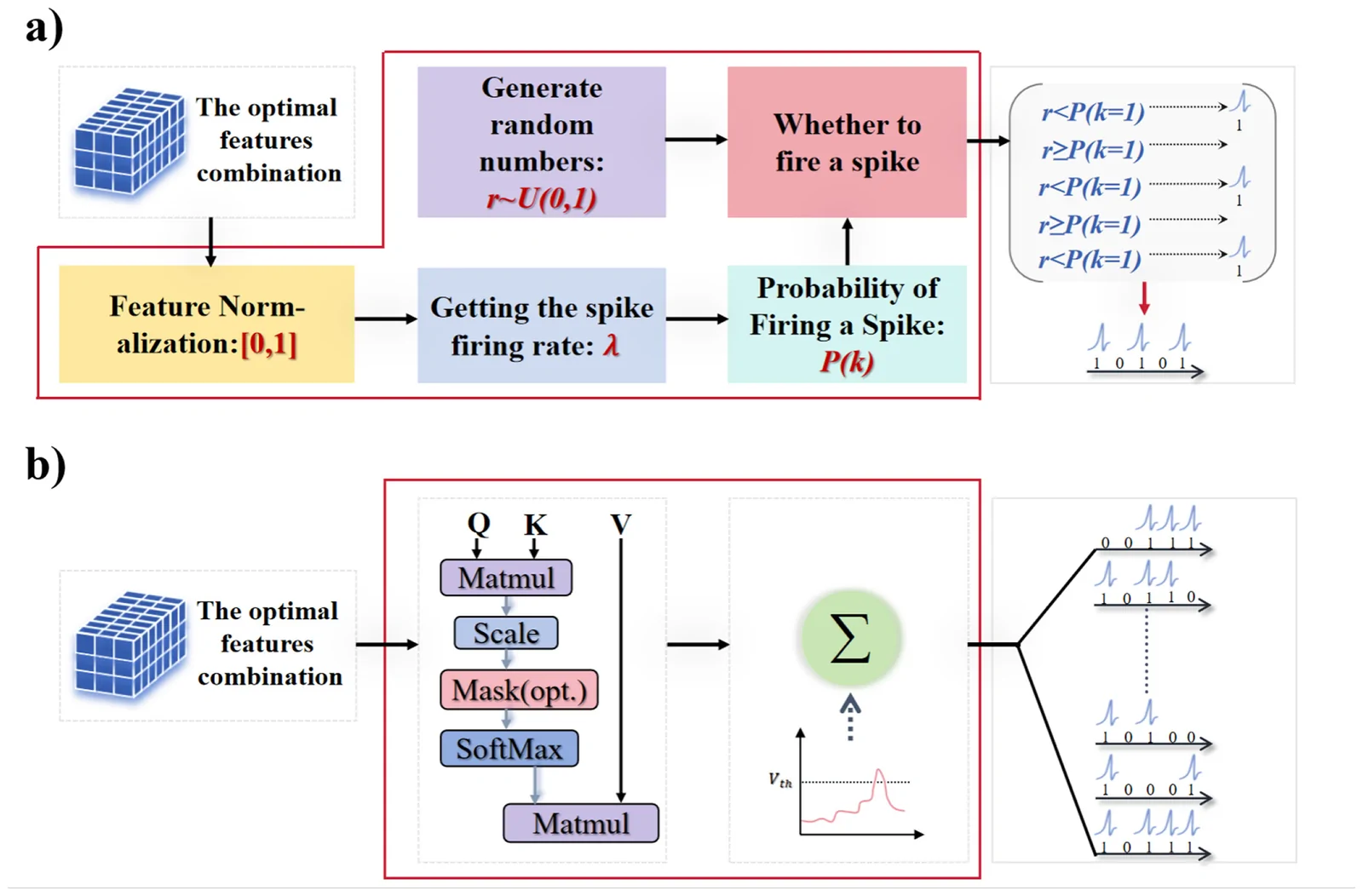
Spike encoding methods. **(a)** Poisson encoding: after normalizing the optimal time-frequency features, the spike firing rate is calculated, and a random number is generated to determine whether a spike fired; **(b)** Improved spike encoding methods: the optimal time-frequency features capture dependencies between time steps through the self-attention mechanism, and a single neuron generates spike sequences that better reflect biological neural activity.

Poisson encoding is a method for converting continuous signals into discrete spike sequences (Auge et al., 2021). It mimics the process by which biological neurons fire spikes in response to varying input signal strengths, as shown in Figure 5a.

Feature normalization: normalization aims to scale the signal values to the [0,1] range, facilitating subsequent processing. The normalization formula is as follows (Equation 25):


$$\begin{array}{l} X_{norm}=\frac{X-X_{min}}{X_{max}-X_{min}} \end{array}$$ (25)

*X*_*norm*_ is the normalized signal value; *X* is the original time-frequency feature value; *X*_*min*_ and *X*_*max*_ are the minimum and maximum values of the signal, respectively.

Getting the spike firing rate: the core idea of Poisson encoding is that the stronger the signal, the higher the probability of firing a spike. This firing rate controls the spike firing frequency. The formula for calculating the spike firing rate is as follows (Equation 26):


$$\begin{array}{l} \lambda =k\cdot x_{\text{norm}} \end{array}$$ (26)

where λ is the spike firing rate, and *k* is a scaling factor used to adjust frequency. The firing rate λ is a probability parameter that controls the intensity of spike firing at each time step. Its value usually ranges between [0, 1], representing the expected spike frequency at a given time step.

Generate random numbers: the randomness in Poisson encoding arises from generating a random number *r* at each time step to determine whether to fire a spike. The random number *r* typically follows a uniform distribution *U*(0, 1). The random number *r* is generated as follows (Equation 27):


$$\begin{array}{l} r\sim U\left(0,1\right) \end{array}$$ (27)

This means that *r* is randomly generated within the [0, 1] interval, with each possible value having an equal probability of occurrence.

Probability of firing a spike: according to the Poisson distribution, the probability of firing a spike *P*(*k* = 1) (the probability of firing one spike) is given by (Equation 28):


$$\begin{array}{l} P\left(k=1\right)=\lambda e^{-\lambda } \end{array}$$ (28)

where *e*^−λ^ is the exponential decay term from the Poisson distribution.

Whether to fire a spike: at each time step *t*, Poisson encoding decides whether to fire a spike based on the generated random number *r* and the computed spike firing probability *P*(*k* = 1) (Equation 29):


$$\begin{array}{l} s\left(t\right)=\{\begin{array}{cc} 1, & \text{if }r<P\left(k=1\right),\\ 0, & \text{if }r\geq P\left(k=1\right), \end{array} \end{array}$$ (29)

where *s*(*t*) represents the output at time step *t*. If *r* < *P*(*k* = 1), the output is 1 (a spike is fired); if *r* ≥ *P*(*k* = 1), the output is 0 (no spike is fired). By repeating the above process, a spike train is generated at each time step.

Poisson encoding, due to its probabilistic and random nature (Liao et al., 2023; Wallace and Dowe, 2000), fails to accurately capture the intricate dynamics of biological neurons. Additionally, Poisson encoding is ineffective in modeling dependencies across different time steps, overlooking the temporal information embedded in the input signals (Rathi and Roy, 2023). To address these limitations, we employ the spike encoding method based on the self-attention mechanism combined with a single neuron, as shown in Figure 5b. This approach not only captures temporal dependencies but also improves the precision of the encoded signals. The detailed steps of the process are as follows:

Computing Query (Q), Key (K), and Value (V): initially, the input time-frequency feature matrix *X* is linearly transformed into three matrices *Q*, *K*, and *V* for subsequent similarity calculations and weighted summation (Equation 30):


$$\begin{array}{l} Q=XW^{Q}\\ K=XW^{K}\\ V=XW^{V} \end{array}$$ (30)

where *X* is the input time-frequency feature matrix with dimensions *n* × *d*, with *n* being the number of time steps, and *d* the feature dimension for each time step. *W*^*Q*^, *W*^*K*^, and *W*^*V*^ are learnable weight matrices used to generate *Q*, *K*, and *V*, respectively, each with dimensions *d* × *d*_*k*_.

Computing attention weights: next, the dot product between the Query and Key matrices is computed to measure the similarity between time steps. To prevent gradient explosion caused by large values, the result is scaled by $\sqrt{d_{k}}$, followed by applying the Softmax function to normalize the similarities into probabilities, thus forming the attention weight matrix α (Equation 31):


$$\begin{array}{l} \alpha =\text{Softmax}\left(\frac{QK^{T}}{\sqrt{d_{k}}}\right) \end{array}$$ (31)

where $\sqrt{d_{k}}$ is the scaling factor used to prevent numerical instability during calculations.

Weighted summation to generate feature representations: the attention weight matrix α is then applied to the Value matrix *V* through weighted summation, producing a new feature representation matrix *Z*. This process captures the dependencies between different time steps (Equation 32):


$$\begin{array}{l} Z=\alpha V \end{array}$$ (32)

*Z* is the final generated feature matrix that contains the global dependency information of the input signal.

Combine a neuron: the neuron accumulates membrane potential by receiving input features *Z*_*t*_. When the accumulated membrane potential *V*(*t*) exceeds a threshold *V*_*th*_, the neuron fires a spike (outputs 1), and the membrane potential is reset. If the potential does not reach the threshold, no spike is fired (outputs 0). By repeating the above process at each time step, the neuron fires spikes based on the accumulated membrane potential, ultimately generating a spike train. This spike train consists of 0 and 1, where 1 indicates that a spike is fired at a given time step, and 0 indicates no spike is fired.

#### 2.3.2 Integrate-and-fire neuron model

Currently, there are various neuronal models with different levels of complexity for SNNs, such as the Integrate-and-Fire (IF), Leak Integrate-and-Fire (LIF), and Izhikevich (IZ) models (Pietrzak et al., 2023). In this study, the IF neuron model is selected as the foundational node in our framework due to its perfect sub-threshold memory accumulation and high computational simplicity (Qin et al., 2023). While the LIF model introduces a temporal leakage term that may cause information loss for sparse spikes, and the IZ model requires solving complex differential equations that drastically increase training latency, the IF model yields the optimal balance of inference velocity and data fidelity, especially since the essential temporal dependencies have already been priorly structured by our self-attention layer. Crucially, as empirically demonstrated by Ricklin (Ricklin, 2023), there is no statistically significant difference in classification performance between the IF and LIF neuron models in window-based macro-signal spike classification tasks due to the minimal impact of sub-threshold leak dynamics. Therefore, the added parameter complexity of the LIF model yields no practical performance benefits for our specific framework.

**Figure 6:**
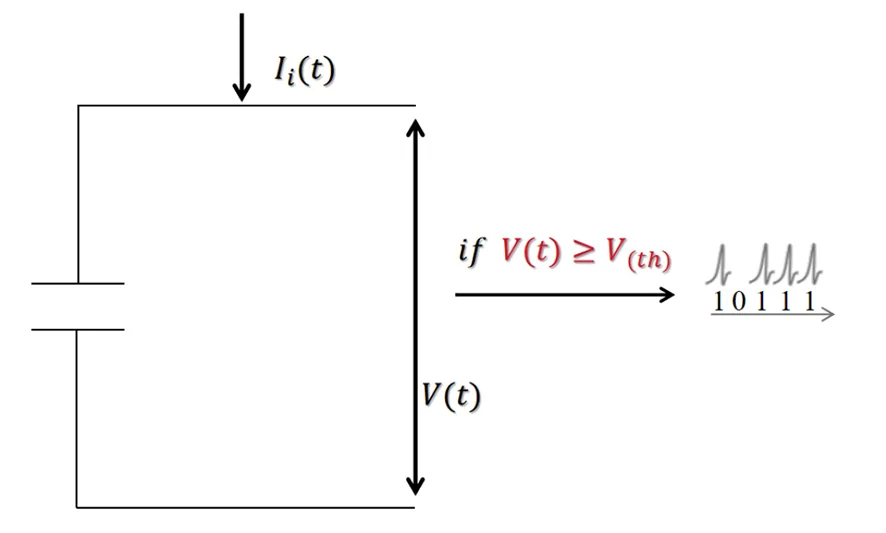
Circuit diagram of the IF neuron model.

The IF neuron model is shown in Figure 6, and the core concept is that the neuron receives input spikes from other neurons, accumulates them internally, and subsequently generates an internal potential.

When the internal potential *V*_*t*_ reaches a set threshold *V*_*th*_, the neuron triggers an output spike that indicates its activation. Subsequently, the internal potential of the neuron is reset to prepare it for the next accumulation and issuance of spikes.

At each time step, the neuron receives a sequence of input spikes from other neurons, which are accumulated into its membrane potential. The membrane potential update can be expressed as (Equation 33):


$$\begin{array}{l} V\left(t\right)=V\left(t-1\right)+\underset{i}{\sum }I_{i}\left(t\right) \end{array}$$ (33)

Here, *V*(*t*) represents the membrane potential at time *t*, *V*(*t*−1) represents the potential at the previous time step, and *I*_*i*_(*t*) denotes the input current from the *i*-th neuron. When the membrane potential *V*(*t*) exceeds the threshold *V*_th_, the neuron fires a spike, and the spike firing process can be described as (Equation 34):


$$\begin{array}{l} \text{if }V\left(t\right)\geq V_{\text{th}}\text{, then emit spike and reset }V\left(t\right)=V_{\text{reset}} \end{array}$$ (34)

After firing, the neuron's internal potential is reset to *V*_reset_, typically the resting potential, preparing it for the next cycle of potential integration and spike firing.

## 3 Experiment

### 3.1 Participants

This study recruited 15 students (12 males, three females; mean age: 21.2 ± 1.5 years) from Changchun University of Science and Technology, all of whom had normal or corrected-to-normal vision. Participants were screened for stereoscopic sensitivity, color vision, and dry eye syndrome. Additionally, they were evaluated for any medical contraindications, including severe concomitant diseases and a history of alcoholism. Before the formal experiment, participants were thoroughly briefed on the experimental procedures. The study involving human participants was reviewed and approved by the Institutional Review Board (IRB) / Ethics Committee of Changchun University of Science and Technology, and conducted in accordance with the Declaration of Helsinki, with informed consent forms signed by all participants.

### 3.2 EEG experiment

**Figure 7:**
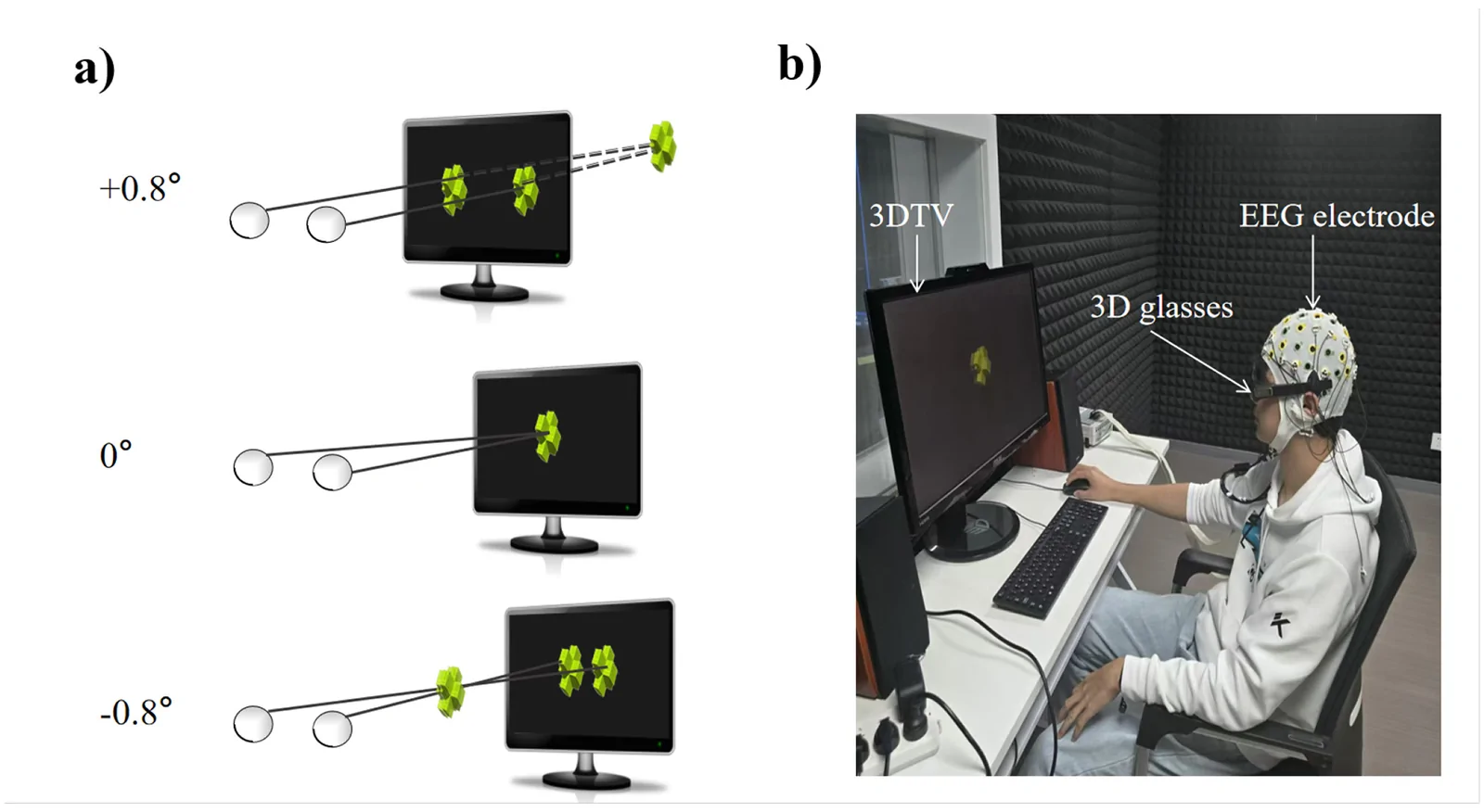
Stereoscopic imaging principle and experimental setup. **(a)** the imaging principle of stereoscopic images, using +0.8°, 0°, and –0.8° parallax values as examples; **(b)** experimental equipment and environment.

The experimental stimulus is stereoscopic image of the superposition of multiple cubes made by unity3D, as shown in Figure 7a. These stereoscopic images formed nine different parallax values of 0°, ±0.3°, ±0.5°, ±0.8°, and ± 1.0° (the default pupil spacing between the two eyes was 65 mm). Participants wearing active shutter glasses sat 1,020 mm away from a 27-inch high-definition stereoscopic monitor (LCD ASUS VG278HR, 1,920 × 1,080 pixels) to view the experimental stimuli, as shown in Figure 7b.

**Figure 8:**
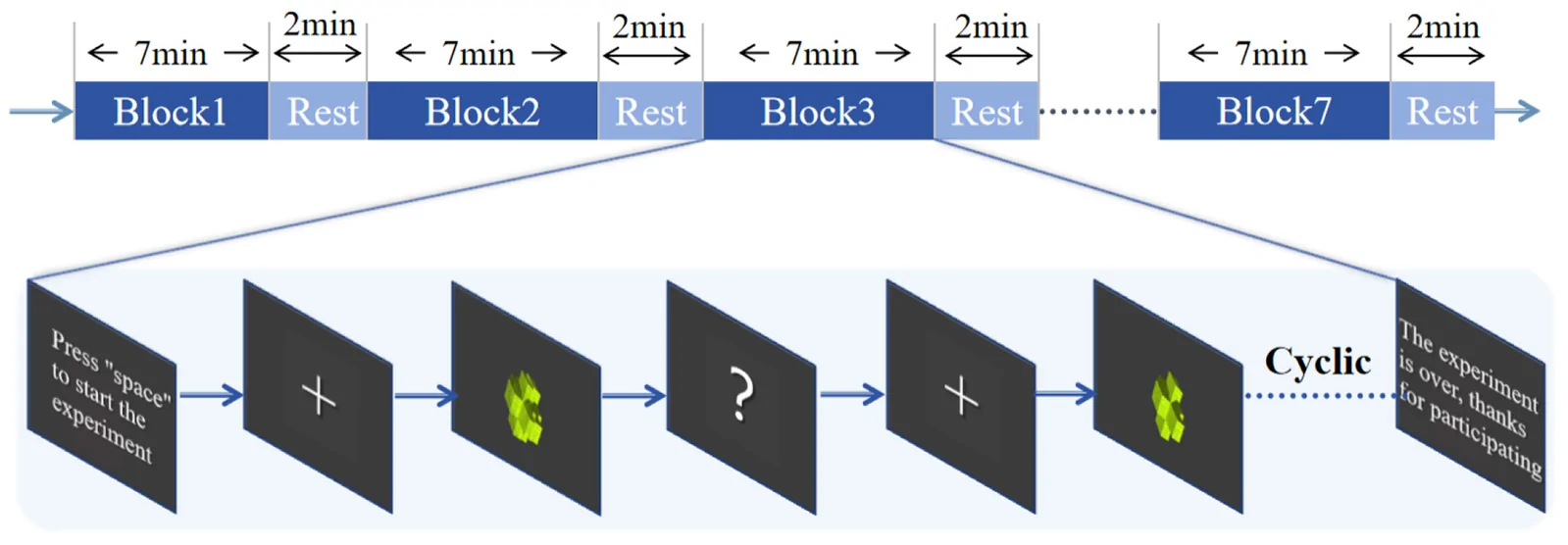
Flow chart of the experiment. Each color represents a different step of the experiment, with 7-min blocks (marked in dark blue) and 2-min breaks (marked in light blue), and the arrows indicate the order in which each step was performed, the viewing process consists of seven blocks.

The experiment was conducted in an electromagnetic shielding room at the Zhongshan Research Institute of Changchun University of Science and Technology to minimize the impact of electromagnetic radiation on physiological signal acquisition. To avoid interference from electronic devices, mobile phones and magnetic cards were not allowed in the laboratory during the experiment. Before the start of the formal experiment, participants performed 5 min pre-experiment to familiarize themselves with the experimental procedure and to confirm that all parallax stereoscopic images were recognized. Participants rested for 5 min to adjust their state to the best condition. The experimental procedure is shown in Figure 8. The screen displays the experiment start interface, the subject presses the “space bar” to start the experiment. Before the start of each trial, a zero-parallax “cross” image was displayed in the center of the screen for 1,000 ms to help subjects focus. Subsequently, one of the parallax images was randomly displayed as the experimental stimulus for 1,000 ms, with each block appearing approximately 15 times for each parallax. At the end of the stimulation, a question mark was displayed in the center of the screen to ask the participants about their state of comfort. If the participant felt visual discomfort (eye pain, nausea, blurred vision), the “left mouse button” was pressed to provide feedback. If no abnormality was felt, the participant waited 1,000 ms for the next stimulus. The experiment was divided into seven Blocks, and when a Block was finished, the participants were allowed to relax and rest for 2 min with their eyes closed before proceeding to the next Block.

### 3.3 Data acquisition

EEG was recorded at a sample rate of 1000 Hz by BrainProducts' 64-channel actiCHamp Plus system. Twenty eight electrodes were positioned and referenced to FCz electrode according to the international 10–20 system, of which 4 electrodes were used to record electrooculogram (EOG) activities and 24 electrodes (FP1, FP2, AF3, AF4, FZ, F7, F8, FC5, FC6, CZ, C3, C4, CP3, CP4, PZ, P3, P4, PO3, PO4, O1, O2, OZ, FT7, FT8) EEG activities were recorded. The impedance of each electrode was kept below 10 kΩ.

### 3.4 Experimental setups

The models in this study were implemented on GeForce 3090 Ti GPUs using Python 3.9 and PyTorch libraries. To improve the generalization ability of the model, a ten-fold cross-validation method was applied to divide the training and test sets rationally. SSA was run for a maximum of 100 iterations to fully extract optimal time-frequency features. The SNN consists of a three-layer architecture: encoding layer, hidden layer, and output layer. In the encoding layer, the spike encoding method based on the self-attention mechanism combined with a single neuron, using 8 attention heads, is employed to capture temporal information from the raw data. The hidden layer contains 100 neurons, each with a threshold of 10 mV. Since this is a binary classification task, the output layer contains two neurons, and a sigmoid activation function is applied to map the output to a probability between 0 and 1. The model parameters were optimized using surrogate gradient descent (Li et al., 2024) and the Adam optimizer (Singanamalla and Lin, 2022), with a learning rate of 0.0002. By minimizing the binary cross-entropy loss function (Zhang et al., 2023), the model was gradually optimized over 100 iterations and achieved good performance in the stereoscopic visual comfort classification task.

## 4 Results and discussion

### 4.1 Effectiveness of feature extraction module

To verify the effectiveness of the feature extraction module in this study, both the features not obtained using this method and those derived from it were classified using machine learning and deep learning models, respectively. The results of this comparison are presented in Table 1. In machine learning, Support Vector Machine (SVM) (Sha'Abani et al., 2020) and Linear Discriminant Analysis (LDA) (Subasi and Ismail, 2010) were used to classify the features obtained using the CSP method, the features obtained in this study, respectively. In deep learning, convolutional neural networks (CNN) (Craik et al., 2019) and transformer (Ahn et al., 2023) are used to classify the features obtained using the CSP method, the pre-processed EEG signals, and the features obtained in this study, respectively. The training and test sets were divided using ten-fold cross-validation. Radial basis kernel function (RBF) was chosen for SVM, while CNN and Transformer were optimized for the training process using Adam with a learning rate of 0.0002.

**Table 1:** Validation of feature extraction validity.

Feature extraction methods	LDA	SVM	CNN	Transformer
CSP	60.32	65.12	85.38	89.63
No feature extraction methods used (raw data)	—	—	80.42	85.61
HHT+CSP+SSA	80.06	84.47	87.34	91.22

CSP: feature extraction using the CSP method; No feature extraction methods used (raw data): the pre-processed EEG signals; HHT+CSP+SSA: the feature extraction module in this study.

The results show that, regardless of using machine learning or deep learning models for classification, the classification accuracy of features obtained by HHT+CSP+SSA method (80.06, 84.47, 87.34, 91.22%) is consistently higher than that achieved with features obtained using the CSP method (60.32, 65.12, 85.38, 89.63%) and raw data (80.42, 85.61%). Moreover, the accuracy of EEG signal classification using deep learning models surpasses that of machine learning models, primarily because deep learning can automatically learn and extract critical features in EEG signals, effectively capturing subtle changes and improving classification accuracy and reliability. Specifically, CNN and Transformer achieved better classification performance with the features obtained by the HHT+CSP+SSA method compared to features extracted using raw data and the CSP method. Compared to raw data, the classification accuracy of CNN and Transformer improved by 7 and 5.6%, respectively. Compared to features extracted using the CSP method, the accuracy of CNN and Transformer increased by 2 and 1.6%, respectively. These results validate that the proposed feature extraction module effectively enhances the performance of the classification models. Therefore, the feature extraction module in this study successfully achieved adaptive extraction of optimal time-frequency features, addressing the limitations of fixed frequency band division and feature redundancy.

Furthermore, regarding the trade-off between the pre-processing complexity and the resulting accuracy margins, it is worth emphasizing that the HHT-CSP-SSA pipeline fundamentally optimizes the global system computing allocation through an offline-online decoupled architecture. While the adaptive time-frequency decomposition and sparrow search algorithm introduce a certain amount of computational overhead during the training and feature selection phase, this process operates strictly offline. By capturing highly discriminative and low-dimensional feature combinations, this approach directly allows the downstream runtime classifier to be exceptionally lightweight, utilizing an SNN with only 100 hidden neurons to achieve an optimal accuracy of 93.86%. In contrast, bypassing the feature extraction module and directly feeding raw EEG data forces the framework to rely on heavy, parameter-dense deep learning models (such as the Transformer backbone), which drastically escalates online deployment latency and computational resource consumption. Therefore, by shifting the primary algorithmic complexity to the offline phase, the proposed framework minimizes real-time inference latency and power consumption on portable edge devices, achieving a globally optimized balance between computational efficiency and classification precision.

### 4.2 Classification results

#### 4.2.1 Performance metrics

The classification performance of the AHCSIS model was comprehensively evaluated by comparing the actual and predicted values using a confusion matrix (Yan et al., 2022). Several metrics were employed to assess the model's performance, including accuracy, precision, F1-score, and kappa coefficient (Virgilio et al., 2020; Weerasinghe et al., 2021; Kim and Kim, 2024). Accuracy represents the proportion of correctly classified samples among all samples. Precision is defined as the proportion of true positives among the samples predicted as positive by the model. The F1-score is the harmonic mean of precision and recall, providing a balanced measure of accuracy and completeness. The kappa coefficient measures the degree of agreement between the model's predictions and the actual classification results. Additionally, the model's performance across different classification thresholds was assessed using the ROC curve (Zhu et al., 2022), which plots the true positive rate (TPR) against the false positive rate (FPR). The formulas for these metrics are as follows (Equations 35–39):


$$\begin{array}{c} \text{Accuracy}=\frac{TP+TN}{TP+TN+FP+FN} \end{array}$$ (35)

$$\begin{array}{c} \text{Precision}=\frac{TP}{TP+FP} \end{array}$$ (36)

$$\begin{array}{c} \text{F1-score}=\frac{2\times \text{precision}\times \text{recall}}{\text{precision}+\text{recall}} \end{array}$$ (37)

$$\begin{array}{c} \text{Kappa}=\frac{p_{o}-p_{e}}{1-p_{e}} \end{array}$$ (38)

$$\begin{array}{c} \text{FPR}=\frac{FP}{FP+TN}\;\text{TPR}=\frac{TP}{TP+FN} \end{array}$$ (39)

**Figure 9:**
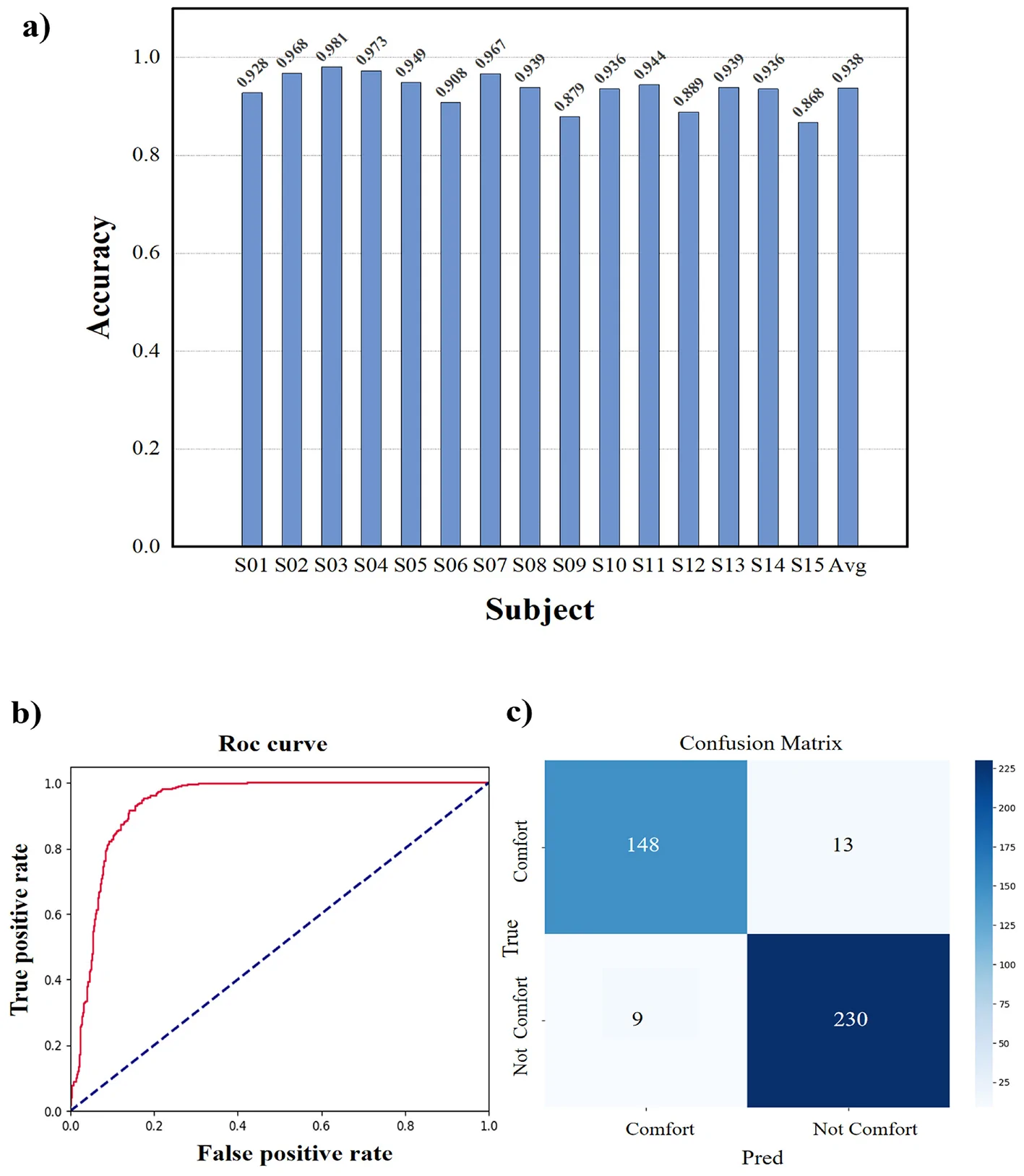
Performance metrics of the AHCSIS model. **(a)** classification accuracy for each subject; **(b)** ROC curve; **(c)** confusion matrix.

Firstly, the horizontal axis of the confusion matrix represents the stereoscopic visual comfort categories predicted by the model, and the vertical axis represents the actual stereoscopic visual comfort categories. It can be seen from Figure 9c that the AHCSIS model achieves good classification results in both visual comfort and discomfort categories. Secondly, as shown in Figure 9a, each participant achieved high classification accuracy on the AHCSIS model, with all individual accuracy above 90% and an average accuracy of 93.86%, demonstrating the model's strong performance. Finally, the AUC value of the ROC curve in the AHCSIS model is about 93%, as shown in Figure 9b, indicating that the curve lies significantly above the diagonal, which demonstrates the model's ability to achieve better TPR and lower FPR across most classification thresholds. As a result, the AHCSIS classification model performs well on several evaluation metrics, and has high classification performance and reliability.

#### 4.2.2 Model comparison

It is compared with baseline and state-of-the-art models to evaluate the performance of the AHCSIS model. A brief description of these models is provided below:

1. S3T: this model uses an attention mechanism to weight the feature channels in the spatial dimension of the data after the CSP method, while perceiving global dependencies along the temporal dimension (Song et al., 2021).
2. NeuroSense: this model achieves efficient and accurate emotion recognition through emotional face detection combined with single-subject SNN classification (Tan et al., 2021).
3. SCNet: this model reduces information loss during spike encoding by embedding CNN, and uses surrogate gradient learning to solve the problem of difficult SNN training (Liao et al., 2023).
4. SGLNet: this model first converts the raw EEG signal into a spike sequence using spike encoding. Then, the intrinsic spatial topological information of the EEG signal is extracted using multi-head adaptive graph convolution. Finally, the temporal dependence is further captured using a long short-term memory network (LSTM) (Gong et al., 2023).

The results comparing the classification performance of various models with the AHCSIS model are presented in Table 2. It can be observed that the NeuroSense, SCNet, and SGLNet models outperform the S3T model. This is likely because the SNN-based models, such as NeuroSense, SCNet, and SGLNet, are primarily designed for tasks involving biological neural activity, whereas the transformer-based S3T model is mainly suited for tasks such as natural language processing and text generation. Compared to the NeuroSense model, the SCNet and SGLNet models show improved performance across all evaluation metrics. This improvement is attributed to the SCNet model's ability to address the information loss problem in spike encoding within SNN, while the SGLNet model further enhances performance by extracting the temporal dependencies of EEG signals using LSTM. However, these improved SNN models do not simultaneously maintain the information accuracy of the original data and the integrity of the timing information when improving certain performances. The spike encoding method based on the self-attention mechanism combined with a single neuron instead of relying on traditional spike encoding, effectively addressing issues of raw data information loss and inference delays in temporal sequences. As a result, the AHCSIS model achieves the highest accuracy, precision, F1-score, and kappa coefficients, demonstrating its exceptional classification performance and reliable capabilities.

**Table 2:** Comparison with the baseline and state-of-the-art models.

Metrics	S3T	NeuroSense	SCNet	SGLNet	AHCSIS
Accuracy	0.9021 ± 0.0124	0.9084 ± 0.0285	0.9153 ± 0.0715	0.9202 ± 0.0981	**0.9386** **±** **0.0475**
Precision	0.9040 ± 0.0542	0.9178 ± 0.1578	0.9045 ± 0.0982	0.9124 ± 0.1012	**0.9312** **±** **0.0124**
F1-score	0.8942 ± 0.0104	0.9205 ± 0.0471	0.9082 ± 0.0310	0.9202 ± 0.1056	**0.9262** **±** **0.0547**
Kappa	0.8932 ± 0.0442	0.9087 ± 0.0525	0.9105 ± 0.0681	0.9233 ± 0.1534	**0.9302** **±** **0.0525**

S3T: attention-based model; NeuroSense: emotion recognition with SNN; SCNet: SNN with CNN embedding; SGLNet: SNN with graph convolution and LSTM. Bold values indicate the best performance achieved in each metric.

#### 4.2.3 Ablation study

The core concept of the AHCSIS model proposed in this study is to adaptively extract time-frequency features from EEG signals using the HHT combined with the CSP method, followed by optimization of the time-frequency feature combination through the SSA. Classification is then performed using the improved SNN. To demonstrate the effectiveness of each component of the AHCSIS model, ablation studies were conducted on all participants, as presented in Table 3.

**Table 3:** Ablation study of the AHCSIS models.

SNN	CSP	HHT	SSA	The self-attention mechanism combined with a single neuron	Accuracy
✓	✓				0.7489 ± 0.0352
✓	✓	✓			0.8650 ± 0.0244
✓	✓	✓	✓		0.8976 ± 0.0182
✓	✓	✓	✓	✓	**0.9386** **±** **0.0475**

Ablation study evaluating the contribution of each component: HHT, Hilbert-Huang transform; CSP, common spatial pattern; SSA, sparrow search algorithm, and the improved spike encoding method (self-attention mechanism combined with a single neuron). Bold values indicate the best performance achieved in each metric.

The spike encoding method based on the self-attention mechanism combined with a single neuron was removed from the AHCSIS model, and classification was performed using the SNN with conventional spike encoding. As shown in Table 3, the results align with our expectations: the average classification accuracy decreased by approximately 5% without the improved spike encoding method. The spike encoding method based on the self-attention mechanism combined with a single neuron effectively captures the informative accuracy and rich temporal information related to stereoscopic visual comfort from the EEG signals. When the SSA was removed from the model, the overall performance decreased by approximately 8%. This verifies that the SSA effectively selects the optimal time-frequency feature combination, contributing significantly to the model's performance. The HHT method was removed to test its contribution to the model, and the results showed that the HHT method can adaptively extract valid time-frequency features in multiple spaces to a large extent, improving the model's average accuracy by about 12%. In summary, the ablation study demonstrates the crucial contributions of each component to the AHCSIS model's overall performance.

## 5 Conclusions

During the feature extraction phase, a combination of HHT and CSP is applied to adaptively extract time-frequency features from EEG. To address the feature redundancy problem, the foraging and anti-predation mechanisms of SSA are used to select the optimal feature combination. In the classification phase, an improved SNN model is used, where the encoding layer applies the spike encoding method based on the self-attention mechanism and a single neuron to classify the extracted features. The contribution of this encoding method lies in enhancing the accuracy and completeness with which the encoding layer represents the dynamic characteristics of biological neural signals, rather than fully simulating biological neural activity in a broad sense. Compared with Poisson encoding, which suffers from information loss and insufficient capture of temporal dependencies due to its stochastic firing mechanism, the proposed method generates spatiotemporal spike sequences that are more faithful to the original signals. Experimental results show that the proposed model outperforms other models in terms of accuracy (93.86%), precision (93.12%), F1-score (92.62%), and kappa coefficient (93.02%). This improvement is attributed to the adaptive feature extraction and the improved spike encoding method. It is demonstrated that the proposed model can be applied to EEG-based classification tasks, providing a novel solution for evaluating stereoscopic visual discomfort.

In conclusion, the AHCSIS model demonstrates robust capability in evaluating stereoscopic visual discomfort based on EEG signals. Future work may focus on developing lightweight models for wearable devices to enable real-time evaluation. Additionally, domain adaptation or transfer learning techniques could be explored to improve cross-subject models, ensuring consistent performance across individual differences while enhancing scalability and practical applicability. Meanwhile, we will continue to refine the model and conduct cross-dataset experimental validation, so as to improve and demonstrate the generalization ability and robustness of the proposed method.

## Data Availability

The raw data supporting the conclusions of this article will be made available by the authors, without undue reservation.

## References

[B1] Agatonovic-Kustrin S. Beresford R. (2000). Basic concepts of artificial neural network (ANN) modeling and its application in pharmaceutical research. J. Pharm. Biomed. Analy. 22, 717–727. doi: 10.1016/S0731-7085(99)00272-1 10815714

[B2] Ahn H-. J. Lee D-. H. Jeong J-. H. Lee S.-W. (2023). Multiscale convolutional transformer for EEG classification of mental imagery in different modalities. IEEE Trans. Neural Syst. Rehabil. Eng. 31, 646–656. doi: 10.1109/TNSRE.2022.3229330 37015688

[B3] Alizadeh D. Omranpour H. (2023). EM-CSP: An efficient multiclass common spatial pattern feature method for speech imagery EEG signals recognition. Biomed. Sig. Proc. Contr. 84:104933. doi: 10.1016/j.bspc.2023.104933

[B4] Anuragi A. Sisodia D. S. Pachori R. B. (2020). Automated alcoholism detection using empirical wavelet transform based features from EEG signals. Biomed. Signal Process. Control 57:101786. doi: 10.1016/j.bspc.2019.101786

[B5] Auge D. Hille J. Mueller E. Knoll A. (2021). A survey of encoding techniques for signal processing in spiking neural networks. Neural Process Lett 53, 4693–4710. doi: 10.1007/s11063-021-10562-2

[B6] Battulga B. Konishi T. Tamura Y. Moriguchi H. (2012). The effectiveness of an interactive 3-dimensional computer graphics model for medical education. Interact. J. Med. Res. 1:e2172. doi: 10.2196/ijmr.2172 23611759 PMC3626131

[B7] Chang H. Zong Y. Zheng W. Xiao Y. Wang X. Zhu J. . (2023). EEG-based major depressive disorder recognition by selecting discriminative features via stochastic search. J. Neural Eng. 20:026021. doi: 10.1088/1741-2552/acbe20 36812637

[B8] Craik A. He Y. Contreras-Vidal J. L. (2019). Deep learning for electroencephalogram (EEG) classification tasks: a review. J. Neural Eng. 16:031001. doi: 10.1088/1741-2552/ab0ab5 30808014

[B9] Delorme A. Makeig S. (2004). EEGLAB: an open source toolbox for analysis of single-trial EEG dynamics including independent component analysis. J. Neurosci. Method 134, 9–21. doi: 10.1016/j.jneumeth.2003.10.009 15102499

[B10] Ford L. K. Borneman J. D. Krebs J. Malaia E. A. Ames B. P. (2021). Classification of visual comprehension based on EEG data using sparse optimal scoring. J. Neural Eng. 18:026025. doi: 10.1088/1741-2552/abdb3b 33440368

[B11] Gad A. G. Sallam K. M. Chakrabortty R. K. Ryan M. J. Abohany A. A. (2022). An improved binary sparrow search algorithm for feature selection in data classification. Neural Comput. Appl. 34, 15705–15752. doi: 10.1007/s00521-022-07203-7

[B12] Gharehchopogh F. S. Namazi M. Ebrahimi L. Abdollahzadeh B. (2023). Advances in sparrow search algorithm: a comprehensive survey. Arch. Comput. Methods Eng. 30, 427–455. doi: 10.1007/s11831-022-09804-w 36034191 PMC9395821

[B13] Ghosh-Dastidar S. Adeli H. (2009). Spiking neural networks. Int. J. Neur. Syst. 19, 295–308. doi: 10.1142/S0129065709002002 19731402

[B14] Gong P. Wang P. Zhou Y. Zhang D. (2023). A spiking neural network with adaptive graph convolution and LSTM for EEG-based brain-computer interfaces. IEEE Trans. Neural Syst. Rehabil. Eng. 31, 1440–1450. doi: 10.1109/TNSRE.2023.3246989 37027669

[B15] Guo S. Wang L. Wang S. Deng Y. Yang Z. Li S. . (2019). “A systolic SNN inference accelerator and its co-optimized software framework,” in Proceedings of the 2019 Great Lakes Symposium on VLSI. 63–68. doi: 10.1145/3299874.3317966

[B16] Higashi H. Tanaka T. (2013). Simultaneous design of FIR filter banks and spatial patterns for EEG signal classification. IEEE Transac. Biomed. Eng. 60, 1100–1110. doi: 10.1109/TBME.2012.2215960 22949044

[B17] Hu H. Pu Z. Li H. Liu Z. Wang P. (2022). Learning optimal time-frequency-spatial features by the CISSA-CSP method for motor imagery EEG classification. Sensors 22:8526. doi: 10.3390/s22218526 36366225 PMC9658317

[B18] Kamble K. S. Sengupta J. (2022). Ensemble machine learning-based affective computing for emotion recognition using dual-decomposed EEG signals. IEEE Sensors J. 22, 2496–2507. doi: 10.1109/JSEN.2021.3135953

[B19] Kang M-. K. Cho H. Park H-. M. Jun S. C. Yoon K.-J. (2017). A wellness platform for stereoscopic 3D video systems using EEG-based visual discomfort evaluation technology. Appl. Ergonomics 62, 158–167. doi: 10.1016/j.apergo.2017.02.022 28411726

[B20] Karatas M. (2008). Central vertigo and dizziness: epidemiology, differential diagnosis, and common causes. Neurologist 14, 355–364. doi: 10.1097/NRL.0b013e31817533a3 19008741

[B21] Kim E. Kim Y. (2024). Exploring the potential of spiking neural networks in biomedical applications: advantages, limitations, and future perspectives. Biomed. Eng. Lett. 14, 967–980. doi: 10.1007/s13534-024-00403-1 39220036 PMC11362408

[B22] Lee P. L. Shang L. Z. Wu Y. T. Shu C. H. Hsieh J. C. Lin Y. Y. . (2009). Single-trial analysis of cortical oscillatory activities during voluntary movements using empirical mode decomposition (EMD)-based spatiotemporal approach. Ann Biomed. Eng. 37, 1683–1700. doi: 10.1007/s10439-009-9730-1 19521773

[B23] Li Y. Fan L. Shen H. Hu D. (2024). HR-SNN: an end-to-end spiking neural network for four-class classification motor imagery brain-computer interface. IEEE Trans. Cogn Dev. Syst. 1–13. doi: 10.1109/TCDS.2024.3395443

[B24] Liao X. Wu Y. Wang Z. Wang D. Zhang H. (2023). A convolutional spiking neural network with adaptive coding for motor imagery classification. Neurocomputing 549:126470. doi: 10.1016/j.neucom.2023.126470

[B25] Liu J. Hu Y. Li G. Pei J. Deng L. (2023). Spike attention coding for spiking neural networks. IEEE Trans. Neural Netw. Learning Syst. 35, 1–7. doi: 10.1109/TNNLS.2023.3310263 37695951

[B26] Ma B. Lu P. Zhang L. Liu Y. Zhou Q. Chen Y. . (2021). Enhanced sparrow search algorithm with mutation strategy for global optimization. IEEE Access 9, 159218–159261. doi: 10.1109/ACCESS.2021.3129255

[B27] Miao Y. Jin J. Daly I. Zuo C. Wang X. Cichocki A. . (2021). Learning common time-frequency-spatial patterns for motor imagery classification. IEEE Trans. Neural Syst. Rehabil. Eng. 29, 699–707. doi: 10.1109/TNSRE.2021.3071140 33819158

[B28] Mishuhina V. Jiang X. (2021).Complex common spatial patterns on time-frequency decomposed EEG for brain-computer interface. Pattern Recogn. 115:107918. doi: 10.1016/j.patcog.2021.107918

[B29] Obata Y. Yamada T. Akiyama K. Sawa T. (2023). Time-trend analysis of the center frequency of the intrinsic mode function from the Hilbert-Huang transform of electroencephalography during general anesthesia: a retrospective observational study. BMC Anesthesiol. 23:125. doi: 10.1186/s12871-023-02082-4 37059989 PMC10105429

[B30] Pietrzak P. Szczesny S. Huderek D. Przyborowski Ł. (2023). Overview of spiking neural network learning approaches and their computational complexities. Sensors 23:3037. doi: 10.3390/s23063037 36991750 PMC10053242

[B31] Qin X. Li C. He H. Pan Z. Lai C. (2023). Python-based circuit design for fundamental building blocks of spiking neural network. Electronics 12:2351. doi: 10.3390/electronics12112351

[B32] Rathi N. Roy K. (2023). DIET-SNN: a low-latency spiking neural network with direct input encoding and leakage and threshold optimization. IEEE Trans. Neural Netw. Learn Syst. 34, 3174–3182. doi: 10.1109/TNNLS.2021.3111897 34596559

[B33] Ricklin S. (2023). Neuron model complexity: exploiting the richness of single neuron dynamics in spiking neural networks. (Master's thesis). Nijmegen: Radboud University.

[B34] Sha'Abani M. Fuad N. Jamal N. Ismail M. F. (2020). “kNN and SVM classification for EEG: a review,” in: *InECCE2019: Proceedings of the 5th international conference on electrical, control and computer engineering* (Singapore: Springer), 555–565. doi: 10.1007/978-981-15-2317-5_47

[B35] Shan X, Huo, S. Yang L. Cao J. Zou J. Chen L. . (2021). A revised hilbert-huang transformation to track non-stationary association of electroencephalography signals. IEEE Trans. Neural Syst. Rehabil. Eng. 29, 841–851. doi: 10.1109/TNSRE.2021.3076311 33909567

[B36] Singanamalla S. K. R. Lin C.-T. (2022). Spike-representation of EEG signals for performance enhancement of brain-computer interfaces. Front. Neurosci. 16:792318. doi: 10.3389/fnins.2022.792318 35444515 PMC9014221

[B37] Song Y. Jia X. Yang L. Xie L. (2021). Transformer-based spatial-temporal feature learning for EEG decoding. arXiv [Preprint]. arXiv:21060.11170.

[B38] Souza U. B. de Escola J. P. L. Brito L. C. (2022). A survey on Hilbert-Huang transform: Evolution, challenges and solutions. Dig. Sig. Proc. 120:103292. doi: 10.1016/j.dsp.2021.103292

[B39] Subasi A. Ismail Gursoy M. (2010). EEG signal classification using PCA, ICA, LDA and support vector machines. Expert Syst. Appl. 37, 8659–8666. doi: 10.1016/j.eswa.2010.06.065

[B40] Tan C. arlija M. Kasabov N. (2021). NeuroSense: short-term emotion recognition and understanding based on spiking neural network modelling of spatio-temporal EEG patterns. Neurocomputing 434, 137–148. doi: 10.1016/j.neucom.2020.12.098

[B41] Tao W. Li, C. Song R. Cheng J. Liu Y. Wan F. . (2023). EEG-based emotion recognition via channel-wise attention and self attention. IEEE Transactions on Affective Computing 14, 382–393. doi: 10.1109/TAFFC.2020.3025777

[B42] Virgilio G. Sossa C. D. Antelis, J. M. Falcn L. E. (2020). Spiking neural networks applied to the classification of motor tasks in EEG signals. Neural Netw. 122, 130–143. doi: 10.1016/j.neunet.2019.09.037 31677441

[B43] Vossen A. Gross J. Thut G. (2015). Alpha power increase after transcranial alternating current stimulation at alpha frequency (α-tACS) reflects plastic changes rather than entrainment. Brain Stimul. 8, 499–508. doi: 10.1016/j.brs.2014.12.004 25648377 PMC4464304

[B44] Wallace C. S. Dowe D. L. (2000). MML clustering of multi-state, Poisson, von Mises circular and Gaussian distributions. Stat. Comput. 10, 73–83. doi: 10.1023/A:1008992619036

[B45] Wang G. Teng C. Li K. Zhang Z. Yan X. (2015). The removal of EOG artifacts from EEG signals using independent component analysis and multivariate empirical mode decomposition. IEEE J. Biomed. Health Informat. 20, 1301–1308. doi: 10.1109/JBHI.2015.2450196 26126290

[B46] Weerasinghe M. M. A. Espinosa-Ramos J. I. Wang G. Y. Parry D. (2021). Incorporating structural plasticity approaches in spiking neural networks for EEG modelling. IEEE Access 9, 117338–117348. doi: 10.1109/ACCESS.2021.3099492

[B47] Wu Y. Feng J. (2018). Development and application of artificial neural network. Wireless Pers Comm. 102, 1645–1656. doi: 10.1007/s11277-017-5224-x

[B48] Xu F. Pan D. Zheng H. Ouyang Y. Jia Z. Zeng H. (2024). EESCN: a novel spiking neural network method for EEG-based emotion recognition. Comput. Methods Prog. Biomed. 243:107927. doi: 10.1016/j.cmpb.2023.107927 38000320

[B49] Xu X. Jia T. Li Q. Wei F. Ye L. Wu X. (2023). EEG feature selection via global redundancy minimization for emotion recognition. IEEE Trans. Affect. Comput. 14, 421–435. doi: 10.1109/TAFFC.2021.3068496

[B50] Yan Z. Zhou J. Wong W.-F. (2022). EEG classification with spiking neural network: smaller, better, more energy efficient. Smart Health 24:100261. doi: 10.1016/j.smhl.2021.100261

[B51] Yang J. Huang D. Zhou D. Liu H. (2020). Optimal IMF selection and unknown fault feature extraction for rolling bearings with different defect modes. Measurement 157:107660. doi: 10.1016/j.measurement.2020.107660

[B52] Yedukondalu J. Sharma L. D. (2023). Circulant singular spectrum analysis and discrete wavelet transform for automated removal of EOG artifacts from EEG signals. Sensors 23:1235. doi: 10.3390/s23031235 36772275 PMC9921497

[B53] Zhang Y. Xu H. Huang L. Chen C. (2023). A storage-efficient SNN-CNN hybrid network with RRAM-implemented weights for traffic signs recognition. Eng. Appl. Artif. Intellig.123:106232. doi: 10.1016/j.engappai.2023.106232

[B54] Zheng J. Liang M. Sinha. S Ge L. Yu W. Ekstrom A. . (2022). Time-frequency analysis of scalp eeg with hilbert-huang transform and deep learning. IEEE J. Biomed. Health Informat. 26, 1549–1559. doi: 10.1109/JBHI.2021.3110267 34516381

[B55] Zheng Y. Zhao X. Yao L. (2022). Copula-based transformer in EEG to assess visual discomfort induced by stereoscopic 3D. Biomed. Sig. Proc. Control 77:103803. doi: 10.1016/j.bspc.2022.103803

[B56] Zhu Z. Lu S. Wang S-. H. Gorriz J. M. Zhang Y.-D. (2022). DSNN: A densenet-based SNN for explainable brain disease classification. Front. Syst. Neurosci. 16: doi: 10.3389/fnsys.2022.838822 35720439 PMC9204288

